# Seasonal Change in Glucose Metabolism and Steroidogenesis in the Ovaries of Wild Ground Squirrels (*Spermophilus dauricus*)

**DOI:** 10.3390/ani16030521

**Published:** 2026-02-06

**Authors:** Zhaomei Dong, Qingjing Gao, Pengyu Chen, Xi Wang, Yuning Liu, Haolin Zhang, Zhengrong Yuan, Yingying Han, Qiang Weng

**Affiliations:** Laboratory of Animal Physiology, College of Biological Sciences and Technology, Beijing Forestry University, Beijing 100083, China; dongzhaomei1919@bjfu.edu.cn (Z.D.); gaoqingjing@bjfu.edu.cn (Q.G.); chenpengyu@bjfu.edu.cn (P.C.); wangyixin808@bjfu.edu.cn (X.W.); yuningliu9@bjfu.edu.cn (Y.L.); haolinzhang@bjfu.edu.cn (H.Z.); zryuan@bjfu.edu.cn (Z.Y.)

**Keywords:** wild ground squirrels, ovary, seasonal breeding, glucose metabolism, steroidogenic enzyme

## Abstract

Precise regulation of ovarian glucose metabolism and steroidogenesis is pivotal for the reproductive success of seasonal breeders. This study investigated seasonal variations in ovarian glucose metabolism and its association with follicular development and steroidogenesis in wild ground squirrels (*Spermophilus dauricus*). Results showed that ovarian weight and volume, and serum levels of luteinizing hormone (LH), follicle-stimulating hormone (FSH), 17β-estradiol, and progesterone were significantly higher in the breeding season (BS) than in the non-breeding season (NBS). Ovarian glycogen content was elevated in the BS, whereas circulating glucose levels decreased remarkably. Histologically, the BS ovaries contained follicles at all developmental stages (primary to Graafian) and corpora lutea, while corpora lutea were absent in the NBS. Integrated transcriptomic and metabolomic analyses revealed upregulated glycogen synthesis, glycolytic pathways, and altered steroidogenic metabolites in the BS. Quantitative real-time PCR (qRT-PCR) confirmed higher expression of glucose transporter 1 (GLUT1), glucose metabolism-related enzymes (G6PD, PFKFB3, PFKM), glycogen synthase (GYS), and steroidogenic factors (StAR, P450scc, P450c17, 3β-HSD, P450arom) in the BS, whereas glycogen synthase kinase-3β (Gsk-3β) was upregulated in the NBS. Immunohistochemistry demonstrated colocalization of steroidogenic and glucose-metabolizing enzymes in granulosa and theca cells, and correlation analyses confirmed positive associations between glucose metabolism, steroidogenesis, and ovarian weight. Collectively, these findings indicate that enhanced ovarian glucose metabolism in the BS is tightly coupled with elevated steroidogenesis, synergistically promoting seasonal follicular development and ovulatory competence in this species.

## Simple Summary

The wild ground squirrel (*Spermophilus dauricus*) is a typical seasonal breeder with distinct cyclical changes in ovarian function and follicular development. During the breeding season (BS), its ovaries contain primary follicles, secondary follicles, antral follicles, Graafian follicles, and corpora lutea; in contrast, only a small number of early-stage follicles exist in the non-breeding season (NBS), with no Graafian follicles or corpora lutea. This pronounced seasonal contrast reflects precise temporal regulation of follicular development and ovulatory activity. Folliculogenesis relies on adequate glucose metabolism (providing energy and essential substrates for follicular growth and oocyte maturation) and steroid hormones (regulating follicular maturation and ovulation). Using integrated multi-omics analyses and experimental validation, our study is the first to reveal that enhanced ovarian glucose metabolism is tightly coupled with elevated steroidogenesis during the BS, synergistically promoting follicular development and ovulatory competence. Conversely, glycogen synthase kinase-3β (GSK-3β)—a negative regulator of glucose metabolism—is upregulated in the NBS, acting as a novel regulatory target to maintain ovarian metabolic quiescence. These findings elucidate the coordinated dynamics of glucose metabolism and steroid hormone synthesis across the seasonal reproductive cycle, providing novel insights into the metabolic–endocrine crosstalk underlying seasonal follicular development in this species and the physiological mechanisms of periodic reproduction.

## 1. Introduction

The precise regulation of glucose metabolism and glycogen synthesis is not only vital for maintaining energy balance but also plays an essential role in various physiological functions. In the reproductive system, adequate glycogen reserves and proper energy supply provide the foundation for germ cell development and endocrine regulation [1,2,3,4]. Glucose metabolism represents the core biochemical pathway for follicular energy acquisition, encompassing a series of physiological processes including glucose uptake, transport, oxidative breakdown, and storage. Glucose is sequentially metabolized via pathways such as glycolysis, the tricarboxylic acid cycle, and oxidative phosphorylation, generating ATP and metabolites including pyruvate and lactate [5,6]. As a hydrophilic molecule, glucose cannot cross the cell membrane; its utilization is instead mediated by a family of glucose transporter proteins (GLUT) [7]. Additionally, glucose exerts a crucial function in follicular development: the pentose phosphate pathway (PPP) generates NADPH and ribose 5-phosphate, both of which are indispensable for nucleotide synthesis [8,9,10]. Glycogen synthesis, a key step in carbohydrate metabolism, is catalyzed by glycogen synthase (GYS) through the glucosyltransferase reaction of UDP-glucose, which forms α-1,4-glycosidic bonds to polymerize into highly branched glycogen molecules [11]. Moreover, glycogen synthase kinase 3 (GSK-3) inhibits glycogen synthesis by phosphorylating and inactivating GYS, thereby fine-tuning the glycogen synthesis process [12]. Studies have confirmed that follicle-stimulating hormone (FSH) can specifically stimulate glycogen synthesis in granulosa cells, and the resultant glycogen reserves provide a critical energy foundation for maintaining granulosa cell function and supporting subsequent steroid synthesis [13].

The generation of a healthy female gamete depends on the coordinated development of granulosa and theca cells within the ovarian follicle, and this coordination requires continuous intercellular communication between these two cellular compartments [14]. Furthermore, follicular development necessitates ovarian steroid hormone production to maintain endocrine homeostasis and support the formation of developmentally competent oocytes, which is a prerequisite for successful fertilization [15]. Steroidogenesis is precisely regulated by steroidogenic enzymes. Specifically, steroidogenic acute regulatory protein (StAR) transports free cholesterol to the inner mitochondrial membrane, where cytochrome P450 side-chain cleavage enzyme (P450scc) converts it to pregnenolone [16]. Pregnenolone is then translocated to the smooth endoplasmic reticulum, where 3β-hydroxysteroid dehydrogenase (3β-HSD) converts it to progesterone, and 17α-hydroxylase/17,20-lyase (P450c17) converts it to dehydroepiandrosterone (DHEA). Both progesterone (via 3β-HSD) and DHEA (via P450c17) are further metabolized to androstenedione, which is subsequently transferred to granulosa cells (GCs). In GCs, cytochrome P450 aromatase (P450arom) converts androstenedione to estrone, and 17β-hydroxysteroid dehydrogenase (17β-HSD) then converts estrone to 17β-estradiol [17]. The synthesized 17β-estradiol exerts diverse physiological effects on ovarian target tissues [18]. Normal glucose metabolism and steroid hormone signaling in GCs are essential not only for oocyte development and maturation but also for the proper growth, differentiation, and functional maintenance of GCs themselves [19].

Seasonal breeding animals provide an ideal natural model for in-depth investigation of follicular development and steroidogenesis, given their inherent periodicity in reproductive activity. The wild ground squirrel (*Spermophilus dauricus*) is a typical seasonal breeder, with its breeding season predominantly occurring from April to May—encompassing mating, pregnancy, parturition, and lactation [16,20,21]. Immediately following the relatively short breeding season, all female individuals—regardless of fertilization status—enter a state of sexual quiescence that persists from June to March of the subsequent year [21,22,23]. Previous research has demonstrated that female wild ground squirrels serve as an excellent model for studying ovarian steroidogenesis and follicular development during the breeding season (BS) and non-breeding season (NBS) [15,20]. Therefore, the present study investigated the expression patterns of genes related to glucose metabolism and steroidogenesis in the ovaries of wild ground squirrels during the BS and NBS. The objectives of this study were to enhance the understanding of seasonal variations in ovarian function in wild ground squirrels and to explore the intrinsic relationships between glucose metabolism, follicular development, and steroidogenesis across the BS and NBS, thereby providing novel insights into the regulatory mechanisms underlying seasonal reproductive adaptation in this species.

## 2. Materials and Methods

### 2.1. Animals and Tissue Preparation

Wild adult female ground squirrels (body weight: 230–419 g) were captured in Hebei Province, China, during the BS (April, n = 6) and the NBS (June, n = 6). All animals were anesthetized with 4% isoflurane and weighed, and blood samples were collected from the saphenous vein of the hind limb. The blood samples (1–1.5 mL) were collected and allowed to clot completely at room temperature. After clotting, the samples were centrifuged at 3000 rpm for 20 min. Subsequently, the serum supernatant (approximately 0.3–0.5 mL) was collected and stored at −80 °C for subsequent analysis. Immediately after the animals were euthanized, the ovaries were collected for analysis. Ovaries from one side of each squirrel (n = 6 per season) were fixed in 4% paraformaldehyde for histological and immunohistochemical examinations. The contralateral ovaries (n = 6 per season) were rapidly frozen in liquid nitrogen and stored at −80 °C for subsequent gene and protein expression analyses. All animal experimental procedures were conducted in compliance with the “Policy on the Care and Use of Animals” approved by the Ethics Committee of Beijing Forestry University (Approval No.: EAWC_BJFU_202008) and authorized by the Hebei Provincial Department of Agriculture (Approval No.: JNZF11/2007).

### 2.2. Glycogen Content Determination

Glycogen content in the ovarian tissues of wild adult female ground squirrels was quantified using the anthrone–sulfuric acid method, following the protocol specified in the Glycogen Content Assay Kit (Solarbio, Beijing, China, Cat# BC0345). Under strongly acidic conditions, glycogen undergoes dehydration to form 5-hydroxymethylfurfural, which then reacts with anthrone to generate blue-green furfural derivatives with a characteristic absorption peak at 620 nm. Glycogen content was calculated in accordance with the manufacturer’s instructions, and the absorbance of each sample was measured using a Bio-Rad microplate reader (Bio-Rad, Hercules, CA, USA).

### 2.3. Histology

Following fixation in 4% paraformaldehyde for 24 h, ovarian tissues were rinsed under running water for 24 h, dehydrated through a graded ethanol series followed by xylene, and subsequently embedded in paraffin. Paraffin blocks were sectioned into 5 μm thick slices, which were floated on a 42 °C warm water bath and then transferred to poly-L-lysine-coated adhesive glass slides to enhance tissue adhesion. After deparaffinization, tissue sections were stained with hematoxylin–eosin (H&E) or Periodic Acid–Schiff (PAS). The stained sections were then dehydrated using a graded ethanol gradient, cleared in xylene, and mounted with neutral balsam under coverslips. Finally, the prepared sections were observed under a light microscope to assess overall tissue morphology, follicle distribution, and cellular composition.

### 2.4. Immunohistochemistry

Following dewaxing, ovarian tissue sections were gently rinsed three times with 0.01 M phosphate buffer (PBS), 5 min per wash. Subsequently, antigen retrieval was performed by incubating the sections in 10 mM citrate buffer (pH 6.0) with heating, followed by natural cooling to room temperature and rinsing as described above. To inactivate endogenous peroxidase activity, the sections were incubated in 3% hydrogen peroxide–methanol solution for 30 min. The sections were then rinsed and blocked with 10% normal goat serum for 1 h at room temperature, followed by incubation with primary polyclonal antibodies (Table 1) at 4 °C overnight (16 h). Following thorough rinsing to remove unbound primary antibodies, the sections were sequentially incubated with biotinylated secondary antibody and horseradish peroxidase (HRP)-conjugated working solution (SP-0022, Bioss Biotechnology, Beijing, China), each for 30 min at room temperature. Signal visualization was achieved with 3,3′-diaminobenzidine (DAB; Wako, Tokyo, Japan) at room temperature, and the reaction was terminated with distilled water. Nuclear counterstaining was performed with hematoxylin, after which the sections were dehydrated, cleared, and sealed with neutral resin. The intensity of DAB-positive signals in ovarian tissues was semi-quantitatively scored using ImageJ software (v1.54d, National Institutes of Health, Bethesda, MD, USA) with the following criteria: − (negative), + (weakly positive), ++ (moderately positive), and +++ (strongly positive) [24].

### 2.5. Blood Hormone and Biochemical Analyses

Serum concentrations of luteinizing hormone (LH), follicle-stimulating hormone (FSH), 17β-estradiol (E_2_), and progesterone (P_4_) were quantified by radioimmunoassay (RIA) using [^125^I]-labeled RIA kits (Beijing Northern Biotechnology Research Institute Co., Ltd., Beijing, China). Each kit had a standard detection range of 5–200 mIU/mL for LH, 2.5–100 mIU/mL for FSH, 0–1000 pg/mL for E_2_, and up to 100 ng/mL for P_4_. The assay sensitivities were approximately 1.0 mIU/mL for LH, <1.0 mIU/mL for FSH, <5 pg/mL for E_2_, and <2 ng/mL for P_4_. The mean recovery rates were 97.0% for LH, 95.9% for FSH, 94–109% for E_2_, and 104.6% for P_4_. Intra- and inter-assay coefficients of variation (CVs) were below 10% and 15%, respectively, for all assays.

Serum glucose concentrations were measured by an external clinical laboratory using a fully automated biochemical analyzer (Toshiba, Tokyo, Japan) and the glucose oxidase (GOD) method, with reagents supplied by Beijing Beijian Xinchuangyuan Science and Technology Co., Ltd. (Beijing, China, Cat# B2007). The assay had a reported linear detection range of 2.20 to 25.00 mmol/L.

All assays were performed in accordance with the manufacturers’ instructions. Results are expressed as follows: LH and FSH in mIU/mL, E_2_ in pg/mL, P_4_ in ng/mL, and glucose in mmol/L. Serum samples were stored at 2–8 °C for up to 48 h or at −20 °C for a maximum of two months, with repeated freeze–thaw cycles avoided.

### 2.6. Total RNA Extraction

Ovarian tissue samples (0.05 g each) were thawed, ground, and lysed on ice. Subsequently, 500 µL of TRIzol reagent was added, and the mixture was incubated on ice for 5 min to ensure thorough lysis. Next, 200 µL of chloroform was added, and the sample was vigorously vortexed for 30 s on ice, followed by incubation undisturbed on ice for 10 min. After incubation, the samples were centrifuged at 12,000× *g* for 15 min at 4 °C. The upper aqueous phase was carefully transferred to a new RNase-free tube on ice, and 500 µL of isopropanol was added to initiate RNA precipitation. The mixture was incubated on ice for 10 min to promote pellet formation. The resulting RNA pellet was washed twice with cold 70% ethanol on ice and then resuspended in 30 µL of DEPC-treated water. RNA integrity was assessed by 1% agarose gel electrophoresis, and RNA concentration was determined using ultraviolet (UV) spectrophotometry. The RNA was then diluted to a final concentration of 500 ng/µL as required for subsequent experiments.

### 2.7. Quantitative Real-Time PCR (qPCR)

First-strand cDNA was synthesized from total RNA using the Hiffair^®^ III 1st Strand cDNA Synthesis Kit (Yeasen Biotechnology, Shanghai, China) for subsequent qPCR analysis. Briefly, in an RNase-free centrifuge tube, 10 μL of RNase-free ddH_2_O, 3 μL of 5× *g* DNA Digester Mix, and 2 μL of total RNA were combined, vigorously mixed with a pipette, and incubated at 42 °C for 2 min to eliminate genomic DNA contamination. Subsequently, 5 μL of 4× Hiffair^®^ SuperMix Plus was added to the reaction mixture, followed by incubation at 25 °C for 5 min, 55 °C for 15 min (cDNA synthesis), and 85 °C for 5 min to terminate the reaction. Following cDNA synthesis, the product was diluted with 80 μL of RNase-free ddH_2_O.

For qPCR, a 10 μL reaction system was prepared by mixing 3 μL of diluted cDNA, 0.1 μL each of forward and reverse primers (100 μM), 5 μL of 2× Power SYBR Green PCR Master Mix, and 1.8 μL of RNase-free ddH_2_O, following the protocol of the FastStart DNA MasterPlus SYBR Green Kit (Roche Molecular Systems Inc., Basel, Switzerland). The sequences of the primers used are listed in Table 2. Real-time PCR was performed on an ABI PRISM 7500 Fast Real-Time PCR System (Applied Biosystems, Foster City, CA, USA). The PCR cycling program included an initial denaturation step at 95 °C for 10 min, followed by 40 cycles of 95 °C for 30 s (denaturation), 60 °C for 30 s (annealing), and 72 °C for 30 s (extension). A melt curve analysis was conducted from 60 °C to 95 °C to verify primer specificity. The relative expression levels of target genes were calculated using β-actin as the internal reference gene, and relative transcription levels were compared via the 2^−ΔΔCt^ method with normalization to β-actin.

### 2.8. Transcriptomic Analysis

Raw sequencing data were subjected to quality control, involving the removal of adapter sequences, tag sequences, reads containing >10% unknown nucleotides, and reads with low-quality bases (Q-value ≤ 20) accounting for more than 50% of the total bases, to generate clean reads. Differentially expressed genes (DEGs) were identified using the DESeq2 (v1.48.2) package in R software (v4.5.0) [25]. DEGs were screened using the criteria of *p* ≤ 0.05 and |Log2FoldChange| ≥ 1. Clustering analysis of DEGs was performed with the Pheatmap (v1.0.13) package to generate a heatmap illustrating gene expression patterns. Gene Ontology (GO) enrichment analysis of DEGs was conducted with Goseq (v1.52.0) software based on the GO database to determine their primary biological functions [26]. Kyoto Encyclopedia of Genes and Genomes (KEGG) pathway enrichment analysis of DEGs was performed using KOBAS (v3.0) software [27] based on the KEGG database [28] to clarify their functional roles in ovarian tissue.

### 2.9. Metabolomic and Multi-Omics Data Analyses

Ovarian tissue samples were submitted to Beijing Kaitai-bio Technology Co., Ltd., (Beijing, China) for non-targeted metabolomic sequencing and subsequent data annotation. Metabolite identification was achieved by matching molecular characteristic peaks against high-resolution mass spectrometry (HRMS) secondary spectral databases (mzCloud, mzVault) and primary mass spectral databases (MassList). Peak extraction was performed with predefined parameters (including ppm tolerance, signal-to-noise ratio [S/N], and total ion current) concurrent with peak area quantification. Metabolite abundance data were subjected to Principal Component Analysis (PCA) for dimensionality reduction, and metabolic pathway annotation was performed using the KEGG database. Both PCA and Orthogonal Partial Least Squares Discriminant Analysis (OPLS-DA) were conducted on the cloud platform provided by Beijing Kaitai-bio Technology Co., Ltd. (https://kaitai.cloud/tools; accessed on 16 January 2025). Statistical analyses were carried out using R software (v4.5.0). Differentially abundant metabolites (DAMs) were identified based on the criteria of *p* < 0.05 and variable importance in projection (VIP) >1. Volcano plots and heatmaps were generated using the ggplot2 (v3.4.3) and Pheatmap (v1.0.13) packages in R, respectively. Integrated KEGG pathway enrichment analysis of metabolomic and transcriptomic data was performed using MetaboAnalyst software (v6.0) [29].

### 2.10. Western Blotting

Next, 30 μg of protein extract from ovarian tissue was mixed with loading buffer. Equal amounts of each sample were separated by 7.5% SDS-PAGE at 80 V and transferred to a PVDF membrane using a wet transfer apparatus (Bio-Rad, Richmond, CA, USA). The membranes were blocked with 3% BSA for 1 h at room temperature, followed by incubation with primary antibodies (Table 1) overnight at 4 °C. After incubation, the membranes were washed three times, each for 5 min, with TBS-T buffer (0.02 M Tris). The membranes were then incubated with the corresponding secondary antibody (1:5000, goat anti-rabbit or goat anti-mouse; Proteintech, Rosemont, IL, USA) for 1 h at room temperature, followed by six washes with TBS-T (5 min each). Protein signals were detected using an enhanced chemiluminescence (ECL) method.

### 2.11. Statistical Analysis

Statistical analyses were performed using GraphPad Prism 10 software (GraphPad Software Inc., San Diego, CA, USA), and statistical significance was assessed via Student’s *t*-test. A *p*-value < 0.05 was considered statistically significant.

## 3. Results

### 3.1. Morphological and Histological Observations

**Figure 1:**
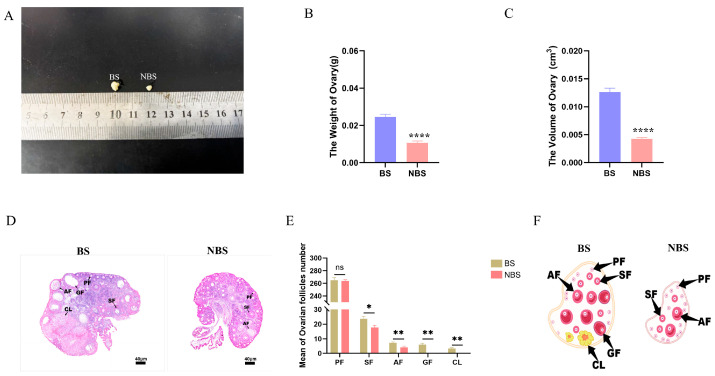
Morphological and histological observations of the ovaries in wild ground squirrels. The morphology observation of the ovary during the BS and NBS (**A**); The weight of the ovaries (**B**); The volume of the ovaries (**C**); The histological observation of the ovaries (**D**); The number of follicles in different stages of development (**E**); The schematic diagrams for the variations in folliculogenesis (**F**). BS, breeding season; NBS, non-breeding season. PF, primary follicle; SF, secondary follicle; AF, antral follicle; GF, Graafian follicle; CL, corpus luteum. Error bars represent mean ± SEM (n = 6 per season). * Indicates statistical significance: * *p* < 0.05, ** *p* < 0.01, **** *p* < 0.0001, ns, not significant. Scale bars represent 40 μm.

Wild female ground squirrels exhibited significant seasonal variations in ovarian morphology and histology (Figure 1). We observed a remarkable reduction in the morphological size of ovaries from NBS squirrels, relative to those from the BS (Figure 1A). Consistently, ovarian weight and volume were markedly elevated during the BS compared with the NBS (Figure 1B,C). Quantitative analysis of HE-stained serial sections further demonstrated significant differences in follicle counts between the two seasons. By contrast, ovaries in the NBS predominantly comprised primordial, primary, and secondary follicles, with antral follicles detected only sporadically. In comparison, ovaries during the BS contained follicles across all developmental stages including primordial, primary, secondary, antral, and Graafian follicles as well as a small number of corpora lutea (Figure 1D,E). Histological pattern diagrams of ovaries during the BS and NBS are presented in Figure 1F.

### 3.2. Seasonal Changes in Serum Levels of LH, FSH, E_2_, and P_4_

**Figure 2:**
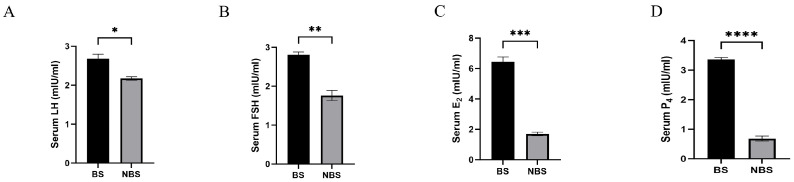
Serum sex hormone concentrations in wild ground squirrels. Serum luteinizing hormone (LH) concentration (**A**); Serum follicle-stimulating hormone (FSH) concentration (**B**); Serum estradiol (E_2_) concentration (**C**); Serum progesterone (P_4_) concentration (**D**). BS, breeding season; NBS, non-breeding season. Error bars represent mean ± SEM (n = 6 per season). * Indicates statistical significance: * *p* < 0.05, ** *p* < 0.01, *** *p* < 0.001, **** *p* < 0.0001.

Serum levels of LH, FSH, E_2_, and P_4_ are presented in Figure 2. Compared with those in the NBS, all these hormone levels were significantly elevated in wild female ground squirrels during the BS (Figure 2A–D).

### 3.3. Seasonal Changes in Serum Glucose Levels, and Ovarian Glycogen Localization and Content

**Figure 3:**
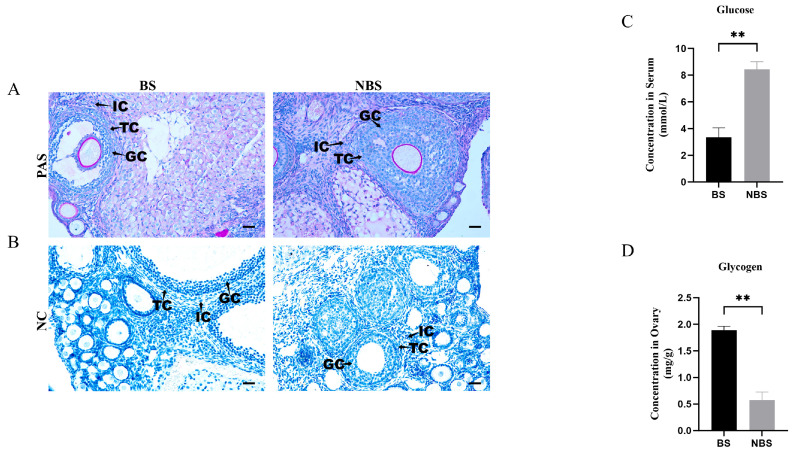
Glycogen localization, serum glucose concentration, and ovarian glycogen levels in wild ground squirrels. Glycogen distribution in the ovary was visualized using PAS staining during the BS and NBS (**A**); Negative controls for the two seasons are shown in the bottom row (**B**); Comparison of serum glucose levels between the BS and NBS (**C**); Comparison of ovarian glycogen levels between the BS and the NBS (**D**). BS, breeding season; NBS, non-breeding season; GC, granulosa cells; TC, theca cells; IC, interstitial cells. Error bars represent mean ± SEM (n = 6 per season). Asterisks indicate statistical significance: ** *p* < 0.01. Scale bars represent 40 μm.

Ovarian glycogen in wild female ground squirrels was localized via Periodic Acid–Schiff (PAS) staining. During the BS, glycogen—appearing purple-magenta—was predominantly concentrated in the cytoplasm of granulosa cells, theca cells, and interstitial cells; it was also detected in these same cell types during the NBS (Figure 3A). No specific signal was observed in the negative controls (Figure 3B). Notably, serum glucose levels were significantly higher in the NBS compared with the BS (Figure 3C). Furthermore, ovarian glycogen content was significantly elevated in the BS relative to the NBS (Figure 3D).

### 3.4. Seasonal Transcriptomic Changes in Ovarian Development: Focus on Glucose Metabolism

**Figure 4:**
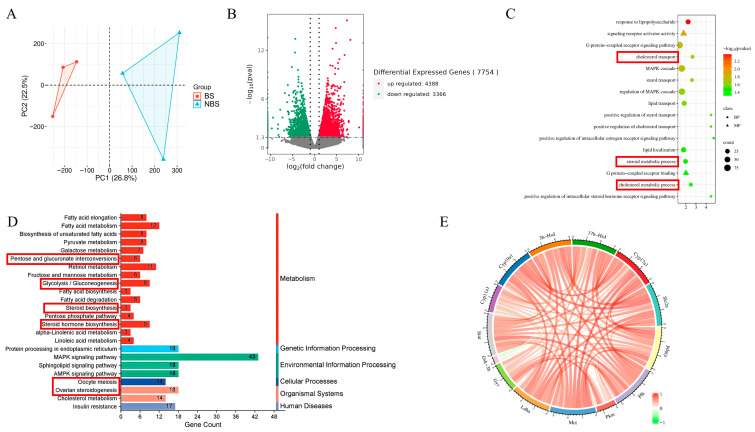
Transcriptomic analysis reveals seasonal changes in ovarian steroid hormone synthesis and glucose metabolism-related gene expression. PCA score plot of ovarian transcriptomes between the BS and NBS (**A**); Number of differentially expressed genes (DEGs) in the BS vs. NBS comparison (**B**); Gene Ontology (GO) enrichment analysis of DEGs (**C**); KEGG pathway enrichment analysis of DEGs (**D**); Pearson correlation circle plot (**E**). BS, breeding season; NBS, non-breeding season.

To investigate the molecular mechanisms underlying glucose metabolism during ovarian development, we profiled the transcriptomes of ovaries from wild ground squirrels in both the BS and NBS. Principal Component Analysis (PCA) of RNA sequencing (RNA-seq) data revealed a clear separation between the two seasonal groups (Figure 4A). Differential gene expression analysis identified 4388 upregulated and 3366 downregulated genes in the NBS relative to the BS (Figure 4B). Gene Ontology (GO) enrichment analysis of differentially expressed genes (DEGs) revealed significant enrichment in biological processes/pathways including the MAPK cascade, steroid metabolic process, cholesterol transport, and glycogen metabolic process (Figure 4C). Notably, these processes were predominantly associated with genes downregulated in the NBS. Kyoto Encyclopedia of Genes and Genomes (KEGG) pathway enrichment analysis showed significant enrichment in glycogen metabolism and steroid hormone metabolism, with key pathways including the MAPK signaling pathway, pentose phosphate pathway, pyruvate metabolism, glycolysis/gluconeogenesis, ovarian steroidogenesis, and oocyte meiosis (Figure 4D). Pathways related to ovarian steroidogenesis and active glucose catabolism were primarily enriched among genes with higher expression in the BS. Notably, ovarian steroidogenesis and glucose metabolism-related pathways were among the most significantly enriched in the ovarian transcriptome (Figure 4D). Additionally, Pearson correlation circle analysis of genes associated with steroid synthesis and glucose metabolism demonstrated a highly significant positive correlation between most of these genes. However, Gsk-3β was an exception, as it exhibited a negative correlation with the other aforementioned genes (Figure 4E).

### 3.5. Seasonal Dynamics of Ovarian Metabolites: Key Roles in Reproductive Mechanisms

**Figure 5:**
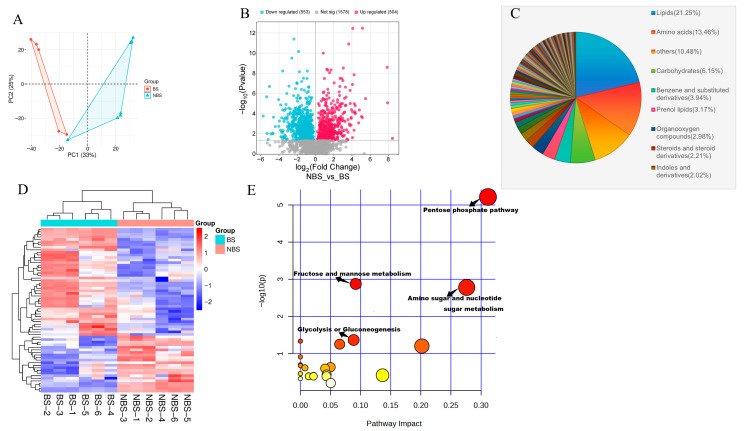
Differential metabolite profiles in the ovary of wild ground squirrels between the BS and NBS. PCA score plot of ovarian metabolomes between the BS and NBS (**A**); Number of differentially abundant metabolites (DAMs) in the NBS vs. BS comparison (**B**); Relative percentage distribution of metabolite classes in the ovarian metabolome (**C**); Heatmap of differentially abundant metabolites (DAMs) (**D**); KEGG pathway enrichment analysis of glucose metabolism-related metabolites (**E**). BS, breeding season; NBS, non-breeding season.

To investigate the effects of seasonal changes on ovarian metabolites in wild ground squirrels, we performed metabolomic profiling of ovarian tissues. Principal Component Analysis (PCA) revealed clear separation between the BS and NBS groups along the first principal component (PC1), which explained 33% of the total metabolic variance (Figure 5A). A total of 2635 metabolites were detected, with 504 upregulated and 553 downregulated in the NBS relative to the BS (Figure 5B). These differentially abundant metabolites (DAMs) were categorized into 172 classes, with lipids as the most abundant class, followed by amino acids, carbohydrates, and steroids (Figure 5C). This finding suggests that lipid and carbohydrate metabolism play pivotal roles in mediating seasonal ovarian metabolic adaptations.

Furthermore, clustering analysis of carbohydrate metabolism-related metabolites revealed distinct seasonal variation patterns between the NBS and BS (Figure 5D). KEGG pathway enrichment analysis of DAMs identified significant seasonal variations in key carbohydrate metabolic pathways. Notably, the pentose phosphate pathway, fructose and mannose metabolism, glycolysis, and glycogen synthesis were significantly enriched among metabolites with higher abundance in the BS (Figure 5E), further supporting the enhanced carbohydrate metabolism in supporting active ovarian function during the breeding season.

### 3.6. Integrated Metabolomic and Transcriptomic Analysis of Ovarian Tissues

**Figure 6:**
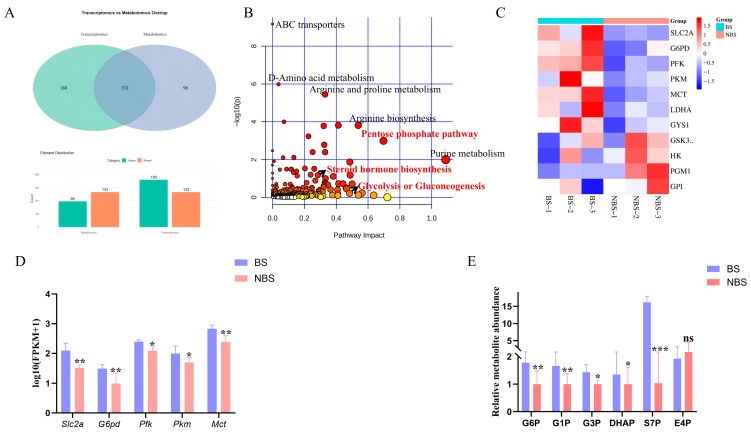
Integrated metabolome and transcriptome analysis reveals dynamic characteristics of glucose metabolism during seasonal changes in wild ground squirrel ovaries. Venn diagram of shared pathways between differentially expressed genes (DEGs) and differentially abundant metabolites (DAMs) in ovaries during the BS and NBS (**A**); Joint-pathway enrichment analysis integrating DEGs and DAMs (**B**); Heatmap of key glucose metabolism-related genes in ovarian transcriptomes (**C**); FPKM levels of key glucose metabolism genes (Slc2a, G6pd, Pfk, Pkm, Mct) in ovaries between the BS and NBS (**D**); Relative levels of key differential metabolites in the glucose metabolism pathway, including G6P (β-D-glucose 6-phosphate), G1P (β-D-glucose 1-phosphate), G3P (glyceraldehyde 3-phosphate), DHAP (dihydroxyacetone phosphate), S7P (D-sedoheptulose 7-phosphate), and E4P (D-erythrose 4-phosphate) (**E**). BS, breeding season; NBS, non-breeding season. * Indicates statistical significance: * *p* < 0.05, ** *p* < 0.01, *** *p* < 0.001, ns, not significant.

To further dissect the molecular and metabolic signatures of ovarian tissues in response to seasonal changes in wild ground squirrels, we performed integrated analyses of transcriptomic and metabolomic datasets. Venn diagram analysis of the multi-omics data revealed 133 pathways shared between the transcriptome and metabolome (Figure 6A). Joint pathway enrichment results showed significant enrichment of ABC transporters, D-amino acid metabolism, arginine and proline metabolism, arginine biosynthesis, and purine metabolism. Notably, the pentose phosphate pathway, steroid hormone biosynthesis, and glycolysis/gluconeogenesis were the most significantly enriched pathways (Figure 6B). FPKM expression levels of glucose metabolism-related genes (Slc2a, G6pd, Pfk, Pkm, and Mct) were significantly upregulated in the ovaries during the BS (Figure 6C,D). Consistently, glucose metabolism-related metabolites in the ovaries were significantly elevated, including G6P (a glycolytic intermediate), G1P (a gluconeogenic intermediate), G3P (involved in both the pentose phosphate pathway and glycolysis), DHAP (a glycolytic intermediate), and S7P (a pentose phosphate pathway intermediate). In contrast, E4P (another pentose phosphate pathway intermediate) exhibited no significant change between the two seasons (Figure 6E).

Collectively, these findings indicate that seasonal alterations in ovarian function are closely associated with dynamic changes in the expression of key glucose metabolism-related genes and the accumulation of corresponding metabolites. These results further validate the utility of wild ground squirrel ovaries as a reliable and physiologically relevant model for investigating the molecular mechanisms underlying follicular development.

### 3.7. Expression of Glucose Metabolism-Related Genes and Proteins

**Figure 7:**
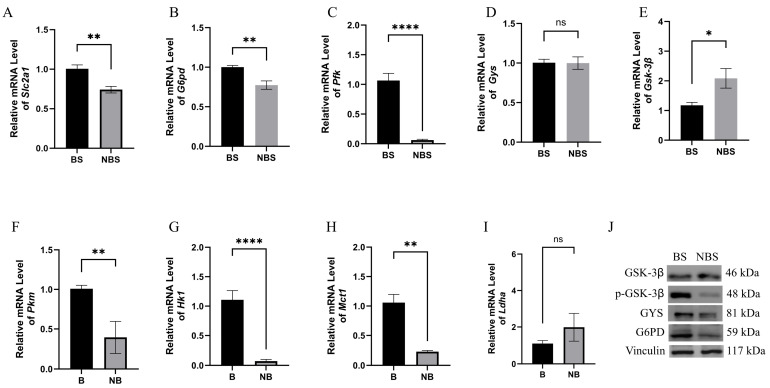
Expression of glucose metabolism-related genes and proteins in wild ground squirrel ovaries. Slc2a1 (**A**), G6pd (**B**), Pfk (**C**), Gys (**D**), Gsk-3β (**E**), Pkm (**F**), Hk1 (**G**), Mct1 (**H**), and Ldha (**I**) mRNA expression levels in ovaries between the BS and NBS, detected by quantitative real-time PCR. The relative proteins levels of GSK-3β, *p*-GSK-3β (ser9), GYS, and G6PD in the ovaries during the BS and NBS (**J**). BS, breeding season; NBS, non-breeding season. Error bars represent mean ± SEM (n = 6 per season). * Indicates statistical significance: * *p* < 0.05, ** *p* < 0.01, **** *p* < 0.0001, ns, not significant.

The mRNA expression levels of Slc2a1, G6pd, Pfk, Gys, and Gsk-3β were quantified in wild ground squirrels during the BS and NBS (Figure 7). Specifically, Slc2a1, G6pd, and Pfk mRNA levels in the ovary were markedly higher during the BS than in the NBS (Figure 7A–C). In contrast, no significant difference in Gys mRNA expression was observed between the two seasons (Figure 7D). Notably, Gsk-3β mRNA expression was significantly lower in the BS compared with the NBS (Figure 7E). Additionally, the mRNA levels of Pkm, Hk1, Mct1, and Ldha were assessed across the two seasons. Consistent with the above results, Pkm, Hk1, and Mct1 mRNA expression in the ovary was significantly elevated during the BS relative to the NBS (Figure 7F–H). However, no significant variation in Ldha mRNA expression was detected between the BS and NBS (Figure 7I). The protein expressions of GSK-3β, *p*-GSK-3β (ser9), GYS, and G6PD in wild ground squirrel ovaries were examined during the BS and NBS (Figure 7J). Specific bands for GSK-3β, *p*-GSK-3β (ser9), GYS, and G6PD were detected at approximately 46 kDa, 48kDa, 81kDa, and 59 kDa, respectively. The protein levels of *p*-GSK-3β (Ser9), GYS, and G6PD were markedly increased in the BS when contrasted with the NBS. Conversely, total GSK-3β levels were elevated in the NBS, indicating that GSK-3β was indeed increased during the non-breeding season, with its protein expression trend consistent with that of its mRNA levels.

### 3.8. Expression of Steroidogenic Enzymes

**Figure 8:**
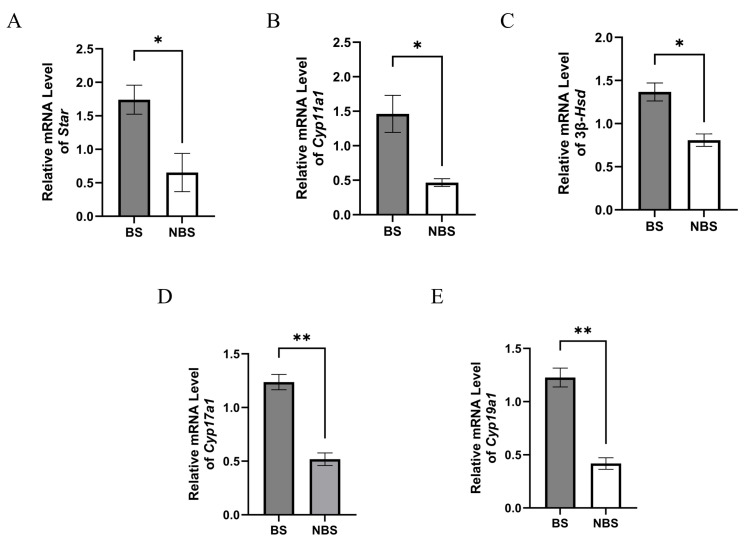
mRNA expression levels of steroid hormone synthesis-related genes in wild ground squirrel ovaries. Star (**A**), Cyp11a1 (**B**), 3β-Hsd (**C**), Cyp17a1 (**D**), and Cyp19a1 (**E**) mRNA expression levels in ovaries between the BS and NBS, detected by quantitative real-time PCR. BS, breeding season; NBS, non-breeding season. Error bars represent mean ± SEM (n = 6 per season). * Indicates statistical significance: * *p* < 0.05, ** *p* < 0.01.

The mRNA expression levels of steroidogenic enzyme-related genes (Star, Cyp11a1, 3β-Hsd, Cyp17a1, and Cyp19a1) were quantified in wild ground squirrels during the BS and NBS (Figure 8). Specifically, Star, Cyp11a1, and 3β-Hsd mRNA levels in the ovary were markedly higher during the BS than in the NBS (Figure 8A–C). Similarly, Cyp17a1 and Cyp19a1 mRNA expression was significantly elevated in the BS relative to the NBS (Figure 8D–E).

### 3.9. Correlation Between mRNA Levels of Glucose Metabolism-Related Genes and Ovarian Weight

**Figure 9:**
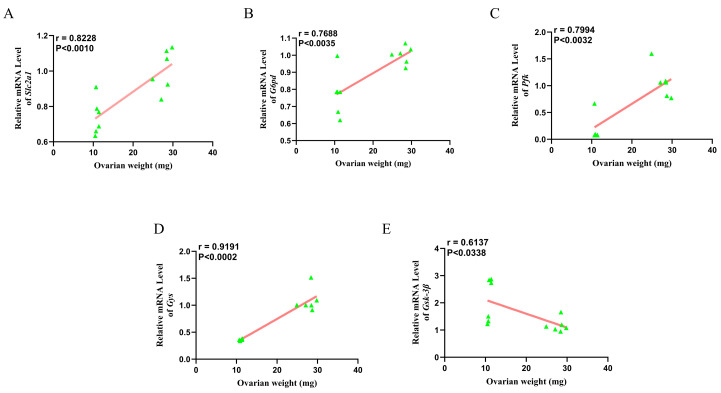
Linear correlation between ovarian weight and relative mRNA expression levels of glucose metabolism-related genes in wild ground squirrels. Correlation between ovarian weight and relative mRNA expression level of Slc2a1 (**A**); Correlation between ovarian weight and relative mRNA expression level of G6pd (**B**); Correlation between ovarian weight and relative mRNA expression level of Pfk (**C**); Correlation between ovarian weight and relative mRNA expression level of Gys (**D**); Correlation between ovarian weight and relative mRNA expression level of Gsk-3β (**E**).

The linear correlation between the relative mRNA expression levels of glucose metabolism-related genes (Slc2a1, G6pd, Pfk, Gys, and Gsk-3β) and ovarian weight in wild ground squirrels during the BS and the NBS is presented in Figure 9. Scatter plots demonstrate that Slc2a1, G6pd, Pfk, and Gys relative mRNA expression levels exhibited a positive correlation with ovarian weight (*p* < 0.05; Figure 9A–D), whereas Gsk-3β relative mRNA expression showed a negative correlation with ovarian weight (*p* < 0.05; Figure 9E).

### 3.10. Correlation Between mRNA Levels of Steroidogenic Genes and Ovarian Weight

**Figure 10:**
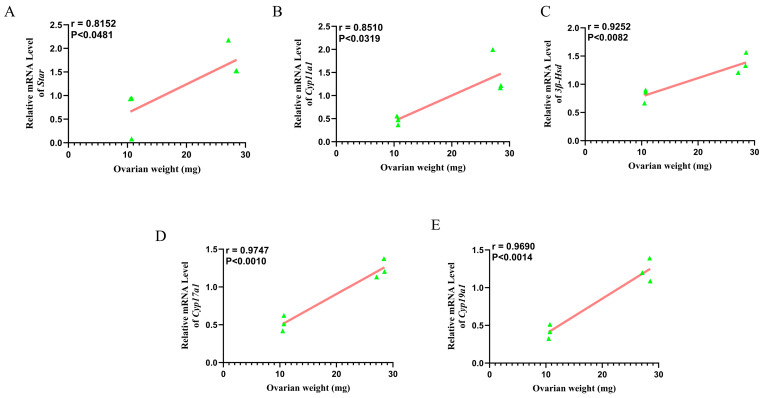
Linear correlation between ovarian weight and relative mRNA expression levels of steroid hormone synthesis-related genes in wild ground squirrels. Correlation between ovarian weight and relative mRNA expression level of Star (**A**); Correlation between ovarian weight and relative mRNA expression level of Cyp11a1 (**B**); Correlation between ovarian weight and relative mRNA expression level of 3β-Hsd (**C**); Correlation between ovarian weight and relative mRNA expression level of Cyp17a1 (**D**); Correlation between ovarian weight and relative mRNA expression level of Cyp19a1 (**E**).

The linear correlation between the relative mRNA expression levels of steroidogenic genes (Star, Cyp11a1, 3β-Hsd, Cyp17a1, and Cyp19a1) and ovarian weight in wild ground squirrels is presented in Figure 10. Scatter plots demonstrate that each of these steroidogenic genes exhibits a significant positive correlation with ovarian weight (*p* < 0.05; Figure 10A–E).

### 3.11. Immunohistochemical Localizations of Glucose Metabolism-Related Proteins

**Figure 11:**
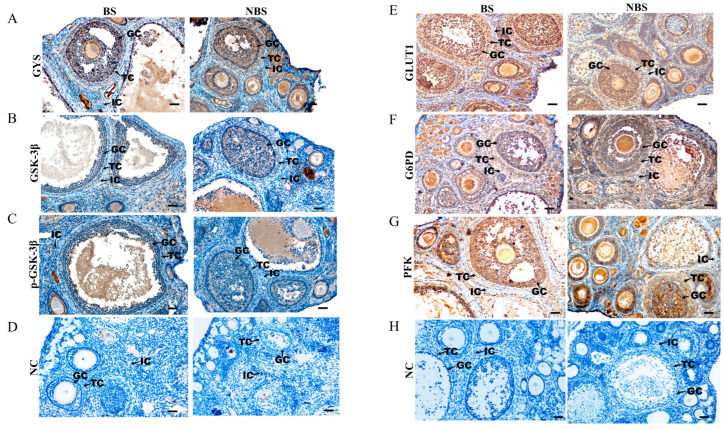
Immunohistochemical localization of glucose metabolism-related proteins in the ovaries of wild ground squirrels. GYS (**A**), GSK-3β (**B**), p-GSK-3β (ser9) (**C**), GLUT1 (**D**), G6PD (**E**), and PFK (**F**) immunolocalization in ovaries during the BS and NBS; Negative controls for the two seasons, shown in the bottom row (**G**,**H**). BS, breeding season; NBS, non-breeding season. GC granulosa cells; TC, theca cells; IC, interstitial cells. Scale bars represent 40 μm.

Immunohistochemical results of GYS, GSK-3β, p-GSK-3β (ser9), GLUT1, G6PD, and PFK in the ovaries of wild ground squirrels are presented in Figure 11. GYS was predominantly localized in the cytoplasm of theca cells, interstitial cells, and granulosa cells during the BS, and was also detected in these same cell types during the NBS (Figure 11A). GSK-3β was localized in the cytoplasm of theca cells, interstitial cells, and granulosa cells in the BS, whereas during the NBS, GSK-3β staining was predominantly observed in granulosa and theca cells (Figure 11B). p-GSK-3β (ser9) was consistently localized in the cytoplasm of both theca cells and granulosa cells throughout both the BS and NBS (Figure 11C). GLUT1 and PFK were expressed in theca cells, interstitial cells, and granulosa cells during both seasons (Figure 11E–G). Notably, G6PD exhibited stronger immunoreactivity in theca and granulosa cells in the BS, with weaker staining observed in the NBS (Figure 11F). No specific signal was detected in the negative controls (Figure 11D,H). The immunohistochemical staining intensity was semi-quantified and is summarized in Table 3.

### 3.12. Immunohistochemical Localization of StAR and Steroidogenic Enzymes

**Figure 12:**
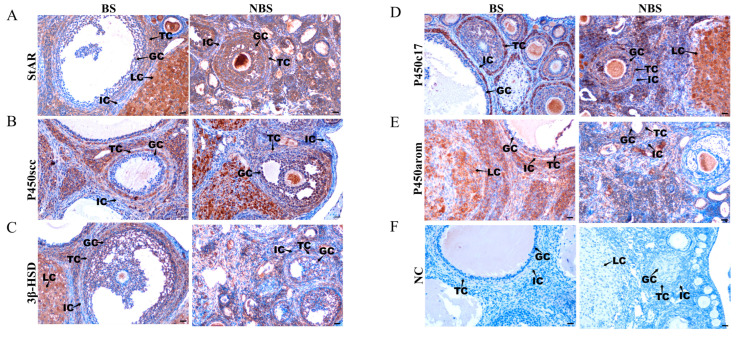
Immunohistochemical localization of steroid hormone synthesis-related proteins in the ovaries of wild ground squirrels. StAR (**A**), P450scc (**B**), 3β-HSD (**C**), P450c17 (**D**), and P450arom (**E**) immunolocalization in ovaries during the BS and NBS; Negative controls for the two seasons shown in the bottom row (**F**). BS, breeding season; NBS, non-breeding season. GC, granulosa cells; TC, theca cells; IC, interstitial cells; LC, luteal cells. Scale bars represent 40 μm.

Immunohistochemical results of StAR and steroidogenic enzymes (P450scc, 3β-HSD, P450c17 and P450arom) in the ovaries of wild ground squirrels during the BS and NBS are presented in Figure 12. StAR, P450scc, and 3β-HSD were predominantly localized in the cytoplasm of ovarian cells during both the BS and NBS (Figure 12A–C). Notably, P450c17 and P450arom exhibited stronger immunoreactivity in theca cells during the BS, while weaker staining was detected in the NBS (Figure 12D,E). No specific signal was observed in the negative controls (Figure 12F). The intensity of immunohistochemical staining was semi-quantified and is summarized in Table 3.

## 4. Discussion

The present study is the first to characterize the expression patterns of genes associated with glucose metabolism and steroidogenesis in the ovaries of wild ground squirrels during the BS and NBS. Our results demonstrated that concomitant with elevated gonadotropin levels and active follicular development, the expression levels of glucose transporter 1 (GLUT1), glycolytic enzymes (GYS1, G6PD, PFKFB3, PFKM), and steroidogenic enzymes (P450scc, P450c17, 3β-HSD, P450arom) were significantly higher during the BS. Glycogen content in ovarian tissues was significantly elevated during the BS, whereas circulating glucose levels decreased remarkably. Consistently, ovarian transcriptomic and metabolomic profiling confirmed that glycogen synthesis, glycolytic pathways, and steroidogenic processes were markedly upregulated in the BS. Collectively, these findings indicate that enhanced ovarian glycolysis is closely correlated with elevated steroidogenesis during the BS, with gonadotropins acting synergistically to support seasonal follicular development and ovulatory capacity in wild ground squirrels.

Beyond molecular signatures, the cyclical alternation between follicular growth and oocyte involution is a well-known phenomenon [30]. In this study, ovarian size and weight were significantly higher during the BS compared with those in the NBS. Meanwhile, primary follicles, secondary follicles, antral follicles, and corpora lutea were identified in ovarian tissues of wild ground squirrels during the BS. These observations are generally consistent with previous reports on this species [15,20] and with observations in other seasonally breeding animals. For example, in female muskrats, ovaries from the breeding season contained primary, secondary, antral, and dominant follicles, while those in the non-breeding season only harbored preantral follicles [31]. A study on raccoon dogs also demonstrated that primary and secondary follicles but no Graafian follicles or corpora lutea were observed in their ovaries during the non-breeding period, whereas various developing follicles and corpora lutea were detected in the breeding period [32]. Similarly, in buffaloes and pigs, seasonal changes in reproductive status affect ovarian follicular development: the form and function of buffalo ovaries are modulated by seasonal reproductive status [33], while porcine oocytes fail to achieve full developmental potential during periods of seasonal infertility [34]. Together with these studies, the present data further provide strong evidence for the notion that follicular development differs between the BS and NBS in seasonally breeding mammals [31].

FSH is a central regulator of ovarian folliculogenesis, and its modulation of GC metabolism, particularly glycolysis, has emerged as a key mechanism driving follicle maturation [35,36]. In ovine GCs, treatment with 10 ng/mL FSH upregulated the transcriptional and protein expression levels of glucose transporters GLUT1 and GLUT4, and induced Akt phosphorylation, with the phosphorylation response peaking at 20 min post-treatment. This phosphorylation event, in turn, regulates FOXO1 phosphorylation, ultimately facilitating glucose uptake [37]. A study on water buffalo GCs directly demonstrated that FSH concentration in follicular fluid correlated positively with glycolytic activity in GCs, as reflected by increased glucose uptake, lactate production, and expression of glycolysis-related genes. In this model, FSH activated the AMPK/SIRT1 pathway to enhance glycolytic enzyme activity, which subsequently supported GC function and follicular development [38]. Consistent with these interspecific observations, our present study showed that during the BS of wild ground squirrels, the expression levels of glucose transporter GLUT1 and glycolytic enzymes (G6PD, PFKFB3, PFKM), and GYS were significantly higher, accompanied by elevated gonadotropin levels, increased ovarian glycogen storage and follicular development in the ovaries. Moreover, our transcriptomic and metabolomic analyses further revealed that upregulated glycogen synthesis, glycolytic pathways, and steroidogenesis represented prominent molecular and metabolic features of the BS. Based on the above findings, FSH enhances GC glycolysis by upregulating critical transporters (GLUT1) and metabolic enzymes, thereby supporting GC proliferation and follicular development. When contextualized with findings from ovine and buffalo GCs, these results reveal that while species-specific signaling cascades may reflect adaptive evolutionary traits, the conserved role of FSH-driven glycolysis underscores the fundamental importance of glucose metabolism in mediating FSH-dependent folliculogenesis across mammalian species.

FSH is a pivotal regulator of ovarian folliculogenesis, and its regulation of steroid hormone synthesis is tightly intertwined with glucose metabolism [39]. Recent studies have unraveled the molecular mechanisms underlying this crosstalk, highlighting the critical role of glycolytic metabolism in FSH-induced steroidogenesis [19,40]. In the present study, the expression levels of StAR, P450scc, P450c17, 3β-HSD, and P450arom were significantly higher during the BS, and these levels were consistently correlated with the concentrations of progesterone (P_4_) and estradiol (E_2_); correspondingly, the concentrations of gonadotropins and the mRNA expression levels of glycolytic enzymes in the ovary were also significantly elevated during the BS. Additionally, our immunohistochemical results revealed the colocalization of steroidogenic enzymes with key glucose-metabolizing enzymes in ovarian follicles, primarily within granulosa and theca cells, collectively suggesting that FSH coordinates glucose metabolism to support steroid hormone production—a process critical for follicle maturation and fertility during the BS. Support for this crosstalk comes from corroborative studies: in vitro and in vivo experiments on mouse GCs confirmed that FSH dose-dependently induces the accumulation of hypoxia-inducible factor-1α (HIF-1α), which in turn activates AMP-activated protein kinase (AMPK) and upregulates GLUT1 expression, significantly enhancing glucose uptake, lactate production, and ATP generation to provide energy for E2 synthesis [19]; furthermore, FSH activates HIF-1 via the PI3K/AKT/mTOR pathway to promote follicular differentiation and the expression of steroidogenic enzymes [41], and additional verification indicates that FSH can stimulate progesterone synthesis in human GCs without inducing luteinization—a process reliant on glucose metabolism for both energy and biosynthetic precursors [40]. Consistent with these mechanisms, earlier research has established that HIF-1α upregulates glycolytic enzymes and glucose transporters such as GLUT1 [42], and these observations align with the present findings that enhanced ovarian glucose metabolism during the BS is closely correlated with elevated steroidogenesis, collectively promoting seasonal follicular development and ovulatory competence in wild ground squirrels.

In contrast to the hormone-driven metabolic activation observed in the BS, the metabolic quiescence of the ovary during the NBS relies on precise regulation by key inhibitory factors. Additionally, the present results showed that GSK-3β/PGSK-3β were immunolocalized in the ovarian granulosa and theca cells. Furthermore, the mRNA level of GSK-3β was upregulated in the NBS, and the protein level of phosphorylated GSK-3β (Ser9), which typically inhibits GSK-3β activity, was significantly elevated during the BS. These changes were accompanied by a significant reduction in ovarian glycogen content and a marked elevation in serum glucose levels compared to the BS. One potential explanation is that GSK-3β may act as a negative regulator of glucose metabolism [43,44], exerting its inhibitory effect by phosphorylating and inactivating glycogen synthase, thereby suppressing glycogen synthesis and restricting overall ovarian glucose metabolic activity [45,46]. This regulatory mechanism is critical for maintaining ovarian “metabolic quiescence” during the NBS, a state characterized by the predominance of early-stage follicles (primordial and primary follicles) and the absence of Graafian follicles or corpora lutea. These findings provide a novel metabolic regulatory target for understanding the molecular basis of follicular development arrest in the NBS, as they potentially link the suppression of glucose metabolism (a core energy and substrate source for follicular growth) to the static state of ovarian folliculogenesis. Previous studies on seasonal breeders have focused primarily on endocrine signaling such as gonadotropin levels or individual steroidogenic pathways in mediating reproductive quiescence during the non-breeding season [31,47,48], but our results suggest a potential pivotal role of Gsk-3β-mediated metabolic suppression as a complementary and equally important regulatory layer.

## 5. Conclusions

The present study provides evidence that enhanced glucose metabolism and steroidogenesis, synergistically regulated by gonadotropins, collectively drive ovarian activation and follicular development during the BS of wild ground squirrels. By identifying Gsk-3β as a potential important mediator of ovarian metabolic quiescence in the NBS, our work fills the gap in understanding the metabolic–endocrine crosstalk underlying seasonal reproduction and provides new insights into the physiological mechanisms of periodic reproduction. Our multi-omics analyses further reveal that the activation of glycolytic and pentose phosphate pathways fulfills the energetic and biosynthetic demands of seasonal reproductive activation, highlighting a potential pivotal role of glucose metabolism in seasonal breeders. Future in vitro studies (e.g., Gsk-3β knockdown/overexpression in granulosa cells, inhibitor treatment combined with FSH/LH stimulation) will further validate the causal link between Gsk-3β-mediated glucose metabolism and follicular development, providing practical insights for modulating seasonal reproductive activity in wildlife and addressing metabolic-related infertility in domestic animals or humans.

## Figures and Tables

**Table 1:** Primary Antibodies Used for Immunohistochemical and Western blot.

Antibody Name	Host Species	Vendor	IHC Dilution	WB Dilution
Anti-GYS	Rabbit	Bioss Biotechnology, Beijing, China (bs-2359R)	1:200	-
Anti-GSK-3β	Mouse	Bioss Biotechnology, Beijing, China (bs-0023M)	1:200	-
Anti-p-GSK-3β	Mouse	Proteintech, Rosemont, IL, USA (67558-1-Ig)	1:1000	-
Anti-Glut1	Mouse	Proteintech, Rosemont, IL, USA (66290-1-Ig)	1:1000	-
Anti-G6PD	Rabbit	Proteintech, Rosemont, IL, USA (25413-1-AP)	1:1000	1:3000
Anti-PFK	Rabbit	Proteintech, Rosemont, IL, USA (13389-1-AP)	1:1000	-
Anti-StAR	Rabbit	Proteintech, Rosemont, IL, USA (12225-1-AP)	1:1000	-
Anti-P450scc	Rabbit	Proteintech, Rosemont, IL, USA (13363-1-AP)	1:1000	-
Anti-P450c17	Rabbit	Proteintech, Rosemont, IL, USA (14447-1-AP)	1:1000	-
Anti-P450arom	Rabbit	Abcam, Shanghai, China (ab18995)	1:1000	-
Anti-3β-HSD	Rabbit	Bioss Biotechnology, Beijing, China (bs-24205R)	1:200	-
Anti-GYS	Rabbit	Proteintech, Rosemont, IL, USA (22371-1-AP)	-	1:5000
Anti-GSK-3β	Rabbit	Proteintech, Rosemont, IL, USA (22104-1-AP)	-	1:5000
Anti-p-GSK-3β(Ser9)	Mouse	Proteintech, Rosemont, IL, USA (67558-1-Ig)	-	1:5000
Anti-Vinculin	Mouse	Proteintech, Rosemont, IL, USA (66305-1-Ig)	-	1:5000

**Table 2:** Oligonucleotide Sequences Used for Quantitative Real-Time PCR.

Gene Name	Primer Sequence (5′-3′)	Primer Sequence (5′-3′)	Product Length (bp)
*Gys*	F:GGTGCTTGACTCTGTTCTGG	R:CAACAAGAGGCTGTGTGGTT	178
*Gsk-3β*	F:GGTGCTTGACTCTGTTCTGG	R:CAACAAGAGGCTGTGTGGTT	176
*Slc2a1*	F:CATGGGCTTCTCGAAACTGG	R:TGAGGATGCCAACAACGATG	182
*G6pd*	F:GGCCGGTGACATCTTCCAC	R:TTGCCATAAGTCAGATCCAGC	150
*Pfk*	F:CAGTCCACTCCACTCCTTCC	R:GGTGGGACGATTATTGGCAG	168
*Pkm*	F:TGGACATCAGATGCTTTGCG	R:CAGAGGTGGAAAATGGTGGC	172
*Hk1*	F:CTCAGCCCCATCTCCATCTC	R:TGTTCGTTTCCTCCTCTCGG	183
*Mct1*	F:TGGTCATGTGGTGTGATCCT	R:TGCGTTTTGGCTTTGGAGAT	199
*Ldha1*	F:GACAGTCCAATGGTCCAGGA	R:TTAAGCTGTCATGGGTGGGT	219
*Star*	F:TCAGCTTCTACCTGTGCCAA	R:GAGGAAGAAGAGGAGGAGGC	194
*Cyp11a1*	F:CATCATATTTGGGGAGCGCC	R:AAAATAGTGTCCCATGCGGC	186
*Cyp17a1*	F:TCTTCTGCTGCTCACCCTAG	R:TGCCCACACGAAAGGAATAG	191
*Cyp19a1*	F:GCACTTGTCTGAATTTCTTGGG	R:CTGGTTACACTTCTGAGGCG	150
*3β-Hsd*	F:GACGGCTTCATACCCCTACA	R:AGGACTCCATTGTTCCCGAG	183
*β-actin*	F:CCTCTATGCCAACACAGTGC	R:CCTGCTTGCTGATCCACATC	206

**Table 3:** Seasonal Immunohistochemical Localization of GYS, GSK-3β, p-GSK-3β, GLUT1, G6PD, PFK, StAR, P450scc, 3β-HSD, P450c17 and P450arom in Wild Ground Squirrel Ovaries.

Antibodies	Breeding Season			Non-Breeding Season		
	GC	TC	IC	GC	TC	IC
GYS	++	+	+	++	++	+
GSK-3β	++	+	+	++	+	−
p-GSK-3β	+	+	−	+	−	−
GLUT1	+	+	+	+	+	+
G6PD	++	++	+	++	+	+
PFK	++	+	+	++	+	+
StAR	+	++	+	+	++	+
P450scc	+	++	+	++	++	+
3β-HSD	++	++	++	+	+	+
P450c17	+	++	+	+	+	+
P450arom	++	++	++	+	+	+

GC, granulosa cells; TC, theca cells; IC, interstitial cells. −, negative staining; +, positive staining; ++, strong positive staining.

## Data Availability

The original contributions presented in this study are included in the article. Further inquiries can be directed to the corresponding authors.
